# An overview of the cycloaddition chemistry of fulvenes and emerging applications

**DOI:** 10.3762/bjoc.15.209

**Published:** 2019-09-06

**Authors:** Ellen Swan, Kirsten Platts, Anton Blencowe

**Affiliations:** 1 School of Pharmacy and Medical Sciences, The University of South Australia, Adelaide, South Australia 5000, Australia; 2 Future Industries Institute, The University of South Australia, Mawson Lakes, South Australia 5095, Australia

**Keywords:** cycloaddition, fulvene, polycyclic scaffolds

## Abstract

The unusual electronic properties and unique reactivity of fulvenes have interested researchers for over a century. The propensity to form dipolar structures at relatively low temperatures and to participate as various components in cycloaddition reactions, often highly selectively, makes them ideal for the synthesis of complex polycyclic carbon scaffolds. As a result, fulvene cycloaddition chemistry has been employed extensively for the synthesis of natural products. More recently, fulvene cycloaddition chemistry has also found application to other areas including materials chemistry and dynamic combinatorial chemistry. This highlight article discusses the unusual properties of fulvenes and their varied cycloaddition chemistry, focussing on applications in organic and natural synthesis, dynamic combinatorial chemistry and materials chemistry, including dynamers, hydrogels and charge transfer complexes. Tables providing comprehensive directories of fulvene cycloaddition chemistry are provided, including fulvene intramolecular and intermolecular cycloadditions complete with reactant partners and their resulting cyclic adducts, which provide a useful reference source for synthetic chemists working with fulvenes and complex polycyclic scaffolds.

## Introduction

**Figure 1:**
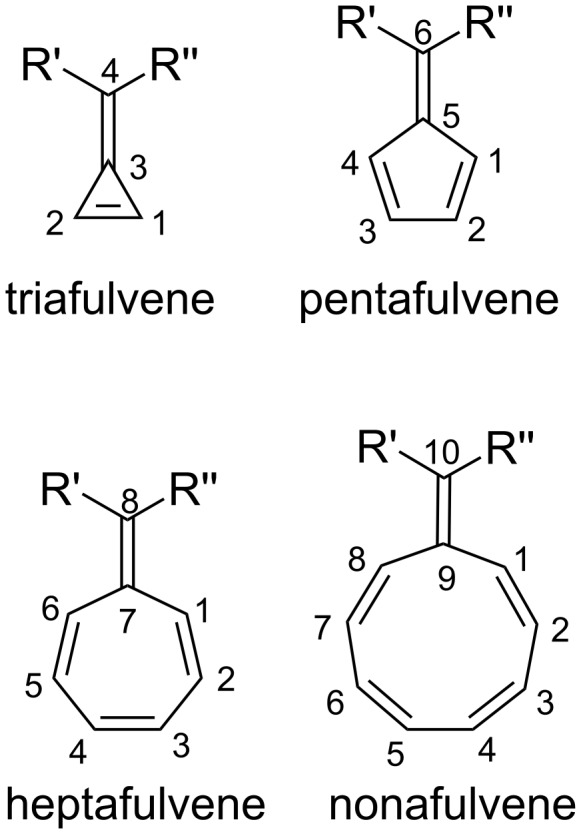
General structure of fulvenes, named according to the number of carbon atoms in their ring. Whilst fulvenes have been numbered using several different systems, *Chemical Abstracts* nomenclature is used throughout this article [34].

Fulvenes are an interesting organic class of cross-conjugated, cyclic molecules first discovered by Thiele in 1900, with the preparation of pentafulvenes by condensation of aldehydes and ketones with cyclopentadiene [1–8]. Most commonly encountered are pentafulvenes, although tria- [4,9–12], hepta- [9,13–28] and nonafulvenes have also been studied (Figure 1). Historically, fulvenes were of great interest as a result of their unique reactivity resulting from their exocyclic double bond [9,29–32], and more recently, as intermediates in the synthesis of more complex polycyclic scaffolds via cycloaddition reactions. While this highlight article will focus primarily on the cycloaddition chemistry of fulvenes and its applications, a brief introduction to the properties and reactivity of fulvenes, important to understanding their participation in cycloaddition reactions, is initially provided. For a more general background on the chemistry of pentafulvenes, in particular their fundamental properties, synthetic transformations, organometallic chemistry and metal-catalysed reactions, an excellent review was recently published by Radhakrishnan and co-workers [33]. This highlight article is intended to give the reader an overview of the varied and exceptional cycloaddition chemistry of fulvenes, and applications that can arise from this.

**Figure 2:**
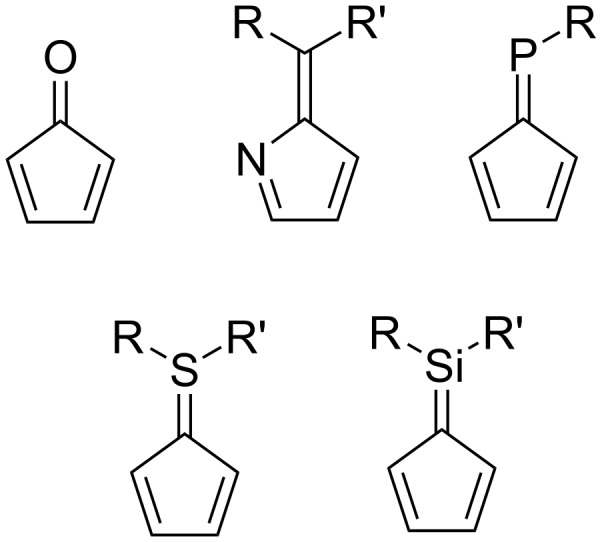
Generic structures of commonly referenced heteropentafulvenes, named according to the heteroatom substitution: oxafulvene, azafulvene, phosphafulvene, thiafulvene and silafulvene.

The replacement of skeletal carbon atoms with heteroatoms affords heterofulvenes. Some common heterofulvenes include oxa-, aza-, sila-, phospha- and thiafulvene derivatives (Figure 2). The introduction of heteroatoms results in differing reactivities, which can be further influenced by substituents, making them useful building blocks for the synthesis of polycyclic compounds [32,35–39]. This is another rich and interesting area of chemistry, although further discussion of heterofulvenes is outside the scope of the current overview and the reader is directed to a very good review by Kawase and Kurata [32].

## Review

### Fulvene properties and reactivity

**Scheme 1:**
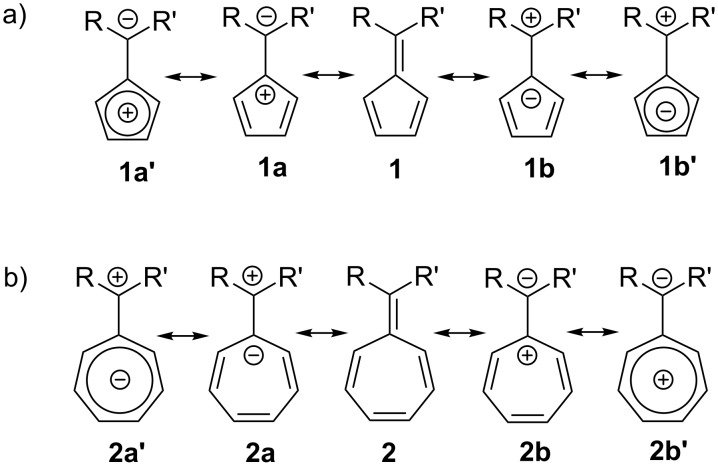
Resonance structures of (a) pentafulvene and (b) heptafulvene showing neutral (**1** and **2**), dipolar (**1a**, **1b**, **2a** and **2b**), aromatic (**1b’** and **2b’**) and anti-aromatic (**1a’** and **2a’**) forms.

The exocyclic double bond of fulvenes is easily polarised, giving rise to dipolar resonance structures (Scheme 1) [1–3,5,9,31–32,40–48].

Generally, fulvenes are thermally unstable, sensitive to oxygen [7,14,49–55], and photosensitive [42,54,56–57]. Fulvenes react with both nucleophiles and electrophiles (according to frontier orbital theory) [1–2,58], and are prone to acid- and cation-catalysed polymerisations [7,14,44,55,58–60]. In addition, fulvenes readily participate in cycloaddition reactions, which will be discussed in more detail in successive sections. The high reactivity of fulvenes is mostly centred about the polarisable exocyclic double bond [1–3,5–6,9,14,32,40,42–45,48,56,61–67]. By considering the dipolar resonance structures of fulvenes, whereby either cationic (**1a** and **2a**) or anionic (**1b** and **2b**) charged centres are formed at the cyclic carbon of the exocyclic double bond, their aromatic character and reactivity becomes more predictable [1–2,5,9,30,42–45,48,52–54,56,62–64,67–69] (Scheme 1).

Whilst delocalisation of electrons from the dipolar form leads to a lower energy aromatic structure for pentafulvene (**1b’**) and nonafulvene derivatives [1,42,45,64,67], similar dipolar forms for triafulvene and heptafulvene (**2a’**) derivatives would lead to higher energy anti-aromatic transition states [1–2,9,15]. Additionally, upon conversion to the dipole forms (Scheme 1), the fulvene loses total planarity through the exocyclic carbon sp^2^ → sp^3^ hybridisation, allowing some loss of energy (and gain in stability) through bond rotation [30,61–62,70].

**Scheme 2:**
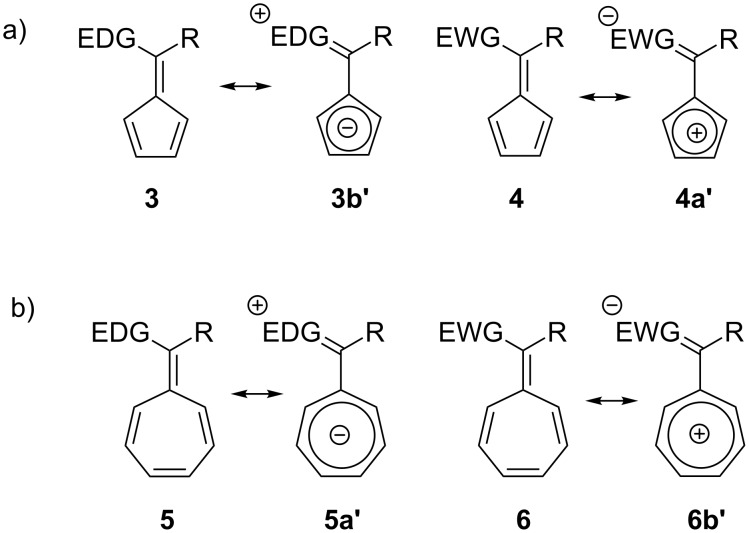
Resonance structures of (a) pentafulvenes and (b) heptafulvenes showing the influence of EDG and EWG on the aromatic (**3b’** and **6b*’***) and anti-aromatic (**4a*’*** and **5a*’***) ring currents.

Furthermore, the nature of the substituents on the exocyclic carbon influences the fulvene reactivity and stability (Scheme 2) [30,42,48,52–54,64,67,69,71]. An ab initio study by Krygowski et al. [15] reported that pentafulvene derivatives (not aromatic in the neutral form), when substituted with electron-withdrawing groups (EWG) (e.g., CN) or electron-donating groups (EDG) (e.g., O, N) on the exocyclic C6-position, exhibited anti-aromatic and aromatic ring currents, respectively [5,29,64,67,72]. Hence, EDG stabilise pentafulvenes (**3b’**), whereas EWG stabilise heptafulvenes (**6b’**) [9,67]. In many cases, the reactions of fulvenes are peri- [17,22,28,73–80], enantio- [81–87], diastereo- [17,21,26,28,81,88–92], and regioselective [17,28,74–76,81–82,93–94], and result from the electronic nature of the fulvene, the reactant partner, as well as steric arguments.

In addition, substituents that are distant, but conjugated to the fulvene group, influence the aromaticity of the molecule [69,71], ultimately allowing modification of the molecule’s reactivity for a given reaction. This was demonstrated in a study by Gugelchuck et al. [71], where the reaction rate of various *p*-substituted 6-phenylpentafulvenes with maleimides was investigated. Substituents of an electron-donating nature (e.g., H, halogens) generally increased the reaction rate through stabilisation of the Diels–Alder transition state, whilst those which were electron-withdrawing (e.g., NO_2_, CN, NMeAc) decreased the reaction rate. Interestingly, strong EDG (e.g., OMe, NMe_2_) exhibited a slower reaction rate than predicted, but this is likely due to the increased stabilisation of the reactant, rather than the transition state [71].

**Scheme 3:**
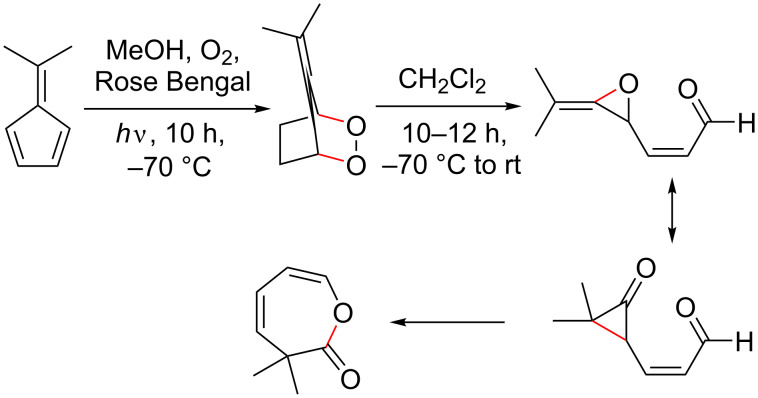
Reaction of 6,6-dimethylpentafulvene with singlet state oxygen to form an enol lactone via the multistep rearrangement proposed by Harada et al. (supporting information was not provided) [51].

Fulvenes can be quite sensitive to oxygen, which has been documented for pentafulvenes and heptafulvenes [16,47,54–55,95]. Pentafulvenes have been reported to react with both ground (triplet) [51–52,55] and excited (singlet) state oxygen [7,49–50,53] resulting in the formation of several different products, although predominantly enol lactones [47,50–52] (Scheme 3). Highly reactive intermediates formed during these reactions (Scheme 3) have only been observed spectroscopically at low temperatures (−55 °C) [52].

**Scheme 4:**
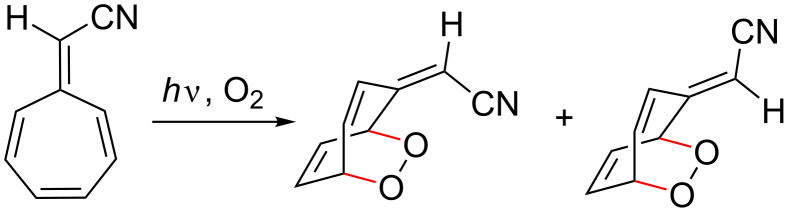
Photosensitized oxygenation of 8-cyanoheptafulvene with singlet state oxygen to afford 1,4-epidioxide isomers [16].

Heptafulvenes also undergo reactions with singlet state oxygen to form similar peroxide, epoxide or epidioxide [16] derivatives, which can be isolated at room temperature (Scheme 4).

**Figure 3:**
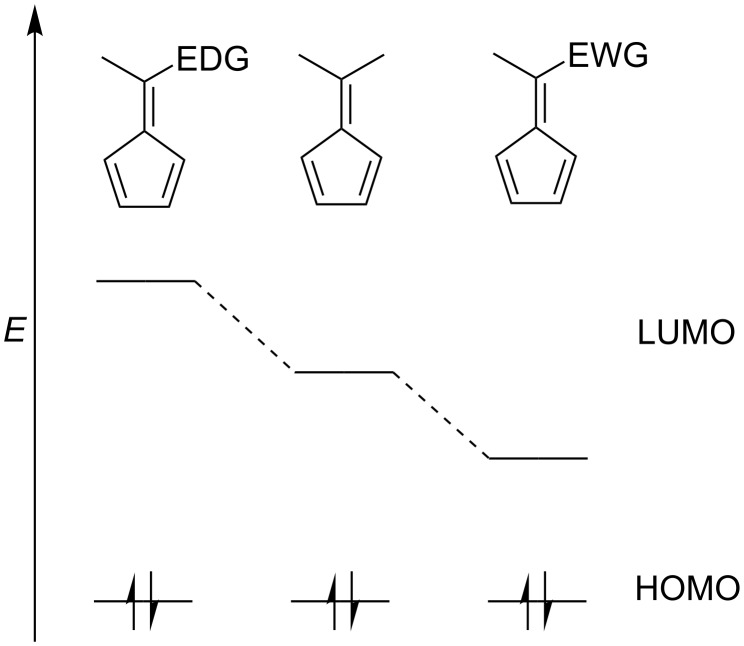
A representation of HOMO–LUMO orbitals of pentafulvene and the influence of EWG and EDG substituents.

An interesting physical characteristic of pentafulvene derivatives is their bright colour, which results from their cross conjugation, and varies with substitution, particularly at the exocyclic C6 position [1–2,6,42,71]. Considering molecular orbital theory, pentafulvenes have a high-energy highest occupied molecular orbital (HOMO) and low-energy lowest unoccupied molecular orbital (LUMO) [1–2,6,42] (HOMO–LUMO) energy gap that is small enough to allow the absorption of long wavelength UV radiation, thus the molecule appears yellow or red [2]. The size of this energy gap can be altered by EWG (−M effect) and EDG (+M effect) substituents (Figure 3), through decreasing or increasing the LUMO energy, respectively [1–3,6–7,42,62,67,71]. In some cases, this can result in a bathochromic shift [2,42]. Consideration of frontier molecular orbital theory allows the electronic nature and general reactivity patterns of fulvenes to be interpreted.

### Fulvene cycloadditions

The multiple cycloaddition pathways observed for fulvenes provides access to a diverse and unique range of fused ring and polycyclic scaffolds. In the subsequent sections, the cycloaddition chemistry of fulvenes will be discussed in terms of their dimerization, and intra- and intermolecular reactions. Whereas the high reactivity and poor stability of triafulvenes have limited studies into their cycloaddition chemistry [1–2,4,10–12], the relative stability of pentafulvenes has allowed extensive research into their participation as 2π, 4π and 6π components. Additionally, pentafulvenes participating as 8π, 10π and 12π components via an extended conjugated chain at the exocyclic C6 position have also been reported. For higher-order hepta- and nonafulvenes, the extended conjugated system also allows them to act as 8π components, as well as 2–6π components.

**Scheme 5:**
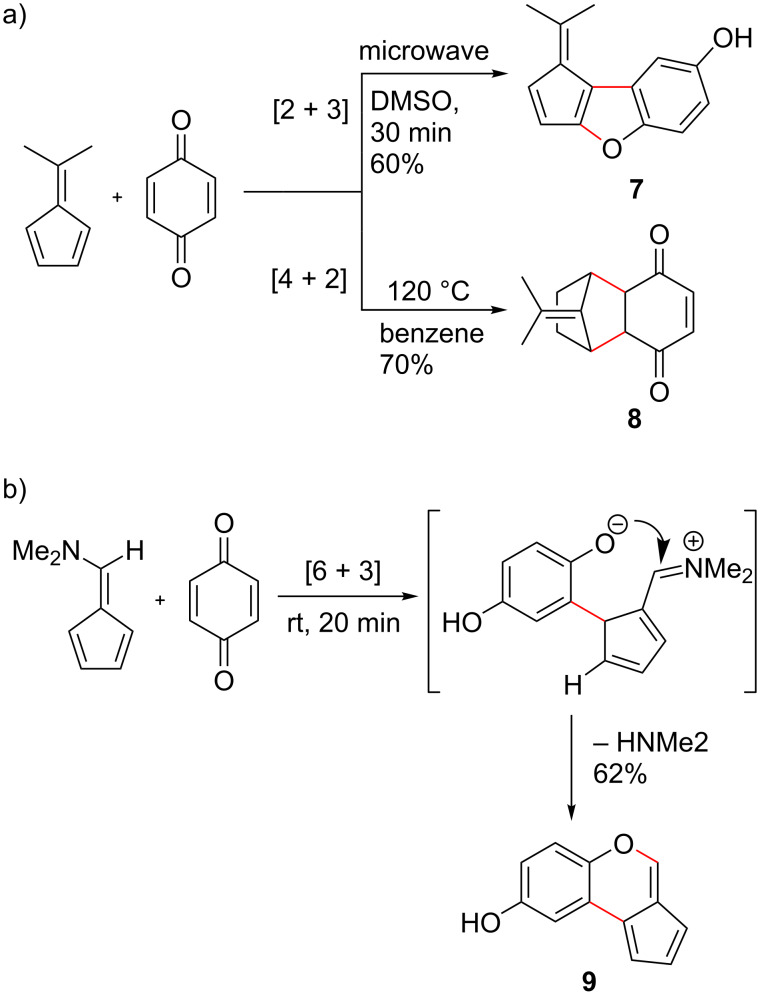
Reactions of (a) 6,6-dimethylpentafulvene participating as 2π and 4π components in cycloadditions with *p*-benzoquinone to afford [2 + 3] (**7**) and [4 + 2] (**8**) cycloadducts, and (b) 6-(dimethylamino)pentafulvene participating as a 6π component in a [6 + 3] cycloaddition with *p*-benzoquinone to afford cycloadduct **9** [32].

Pentafulvenes can react as 2π components with moderately electron-deficient dienes and 4π components in reactions with dienophiles (Scheme 5a), whereas pentafulvenes substituted with EDG (e.g., NMe_2_) at the exocyclic C6 position possess an increased electron density about the fulvene π-system, increasing the stability and hence nucleophilicity of the fulvene [29,73,96–104]. This allows the fulvene to function as a 6π component in reactions with electron-deficient dienes (Scheme 5b) and fulvenes acting as dipolarophiles have been reported for enantioselective [6 + 3] and [3 + 2] cycloadditions [83–84,105]. In general, reactions with electron-rich alkenes will take place preferentially at the exocyclic C6 position while other less electron-rich species interact most strongly with the fulvene HOMO resulting in only [4 + 2] cycloadditions [101,103].

**Scheme 6:**
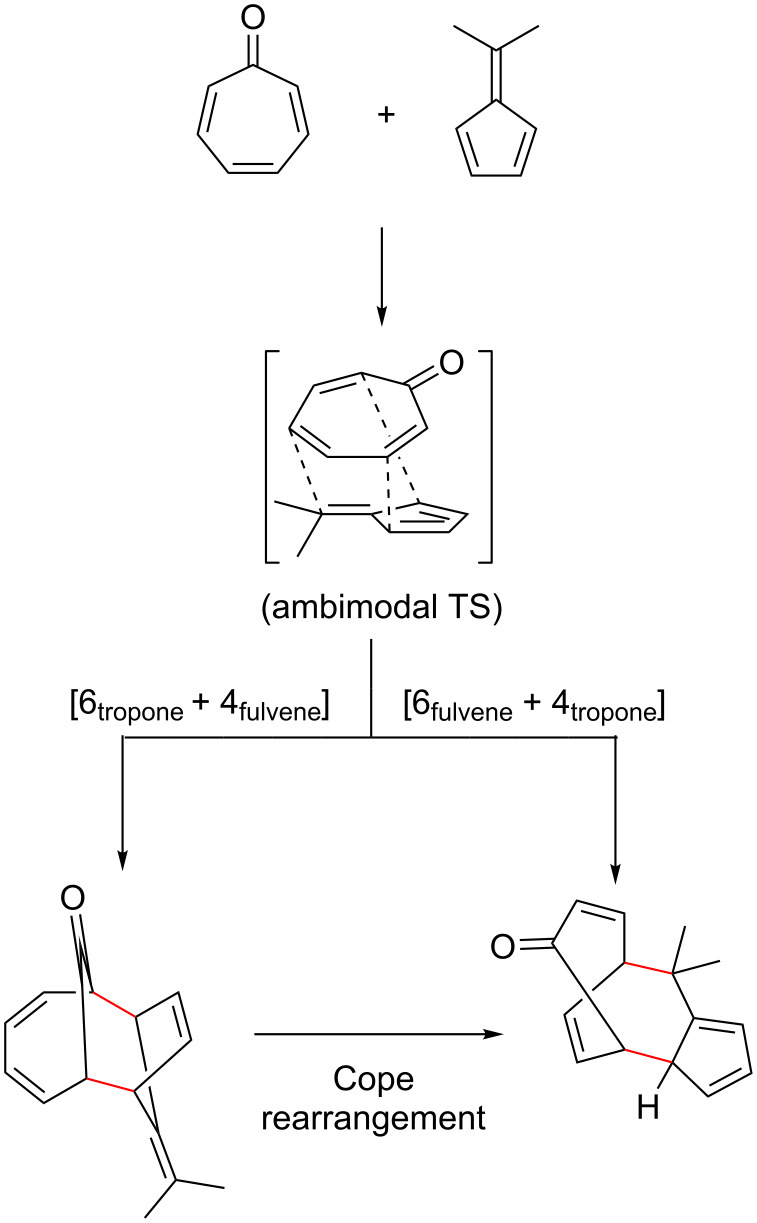
Proposed mechanism for the [6 + 4] cycloaddition of tropone with dimethylfulvene via an ambimodal [6 + 4]/[4 + 6] transition state.

In some cases, cycloaddition reactions involving fulvenes may be difficult to characterise due to the high reactivity of the fulvene group, and the ability to act as multiple cycloaddition components, leading to multiple mechanistic pathways. For example, the cycloaddition of tropone and fulvenes was initially proposed by Houk to proceed via a peri-, regio- and stereoselective [6 + 4] cycloaddition of tropone [4π] to fulvene [6π] [106]. However, an alternate mechanism was proposed by Paddon-Row and Warraner [74], whereby an initial [6 + 4] cycloaddition of tropone [6π] to fulvene [4π] and subsequent Cope rearrangement produced the formal [6 + 4] adduct. More recently, Yu et al. demonstrated through computations that the initial cycloaddition proceeds through an ambimodal [6 + 4]/[4 + 6] transition state leading to both of the proposed [6 + 4] adducts, which can interconvert through a Cope rearrangement (Scheme 6) [107].

### Dimerisation cycloadditions

**Scheme 7:**
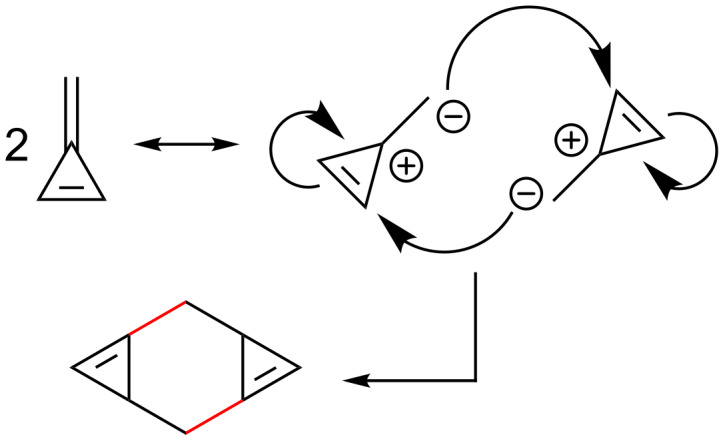
Triafulvene dimerization through the proposed 'head-to-tail' mechanism. The dipolar transition state is shown as the intermediate [4].

Generally, dimerization of fulvenes is an undesired process that may occur upon storage, or compete during reactions with other substrates. As a result of their structure and reactivity, triafulvene [4] and pentafulvene [31,59,66,108–118] derivatives are susceptible to dimerization. The high ring strain of triafulvenes makes them particularly thermally unstable, with dimerization occurring at temperatures higher than −75 °C [4]. The dimerization of triafulvene derivatives is hypothesised to occur via a [4 + 4] cycloaddition pathway (Scheme 7) [4]. Whilst the dimers are also unstable (rapid decomposition when neat), they can be observed spectroscopically at −20 °C [4].

**Scheme 8:**
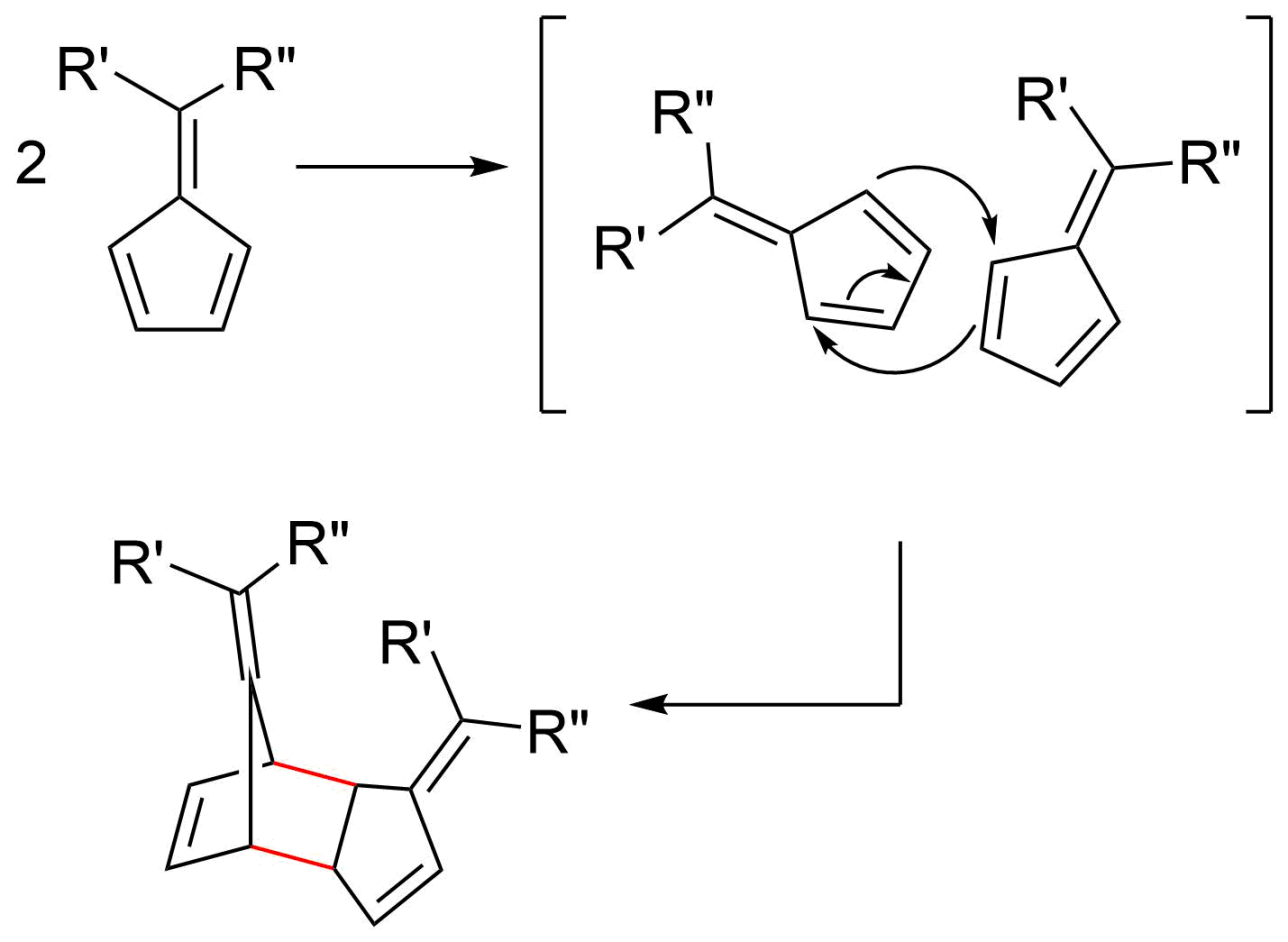
Dimerization of pentafulvenes via a Diels–Alder cycloaddition pathway whereby one fulvene acts as a diene and the second fulvene acts as a dienophile.

There have been numerous reports of pentafulvenes undergoing dimerization via Diels–Alder cycloadditions (DACs) (Scheme 8) at room temperature [6,66,108–109,114–115,117,119]. In some cases, the resulting dimers can undergo subsequent cycloadditions to form trimers via [6 + 4] cycloadditions [109–110] or polymeric products [6,71,109,118], which are often not desired due to the difficulties associated with purification.

**Scheme 9:**
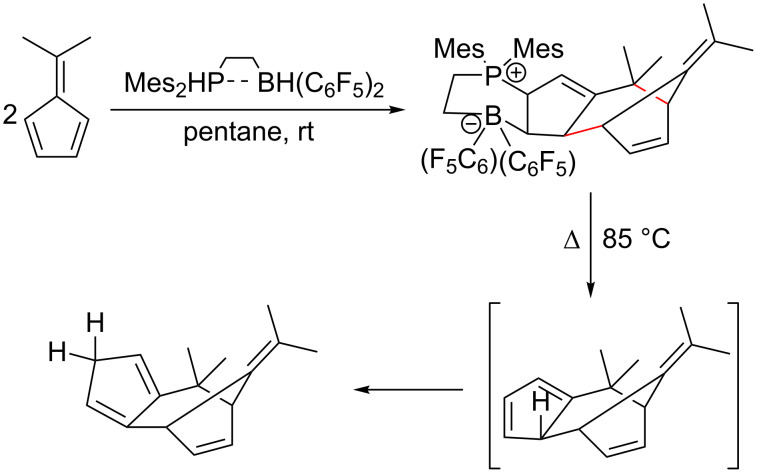
Dimerization of pentafulvenes via frustrated Lewis pair chemistry as reported by Mömming et al. [116].

Additionally, a formal [6 + 4] dimerization was reported by Mömming et al. utilising frustrated Lewis pair chemistry (Scheme 9), however, the mechanism of this process requires further clarification [116].

The rate of dimerization is partly dependent on the fulvene reactivity, which is strongly influenced by its substituents (as discussed previously). For instance, stabilised tria- and heptafulvenes with EWG and penta- and nonafulvenes with EDGs dimerize more slowly [42,64–65,67,71]. The rate of dimerization is also affected by the hydrophilicity and solubility of the fulvene, with groups that lower the hydrophobic character appearing to decrease the rate. For example, the anti-aromatic resonance structure of pentafulvene (**1a’**) (Scheme 1), which is highly reactive, is prone to dimerization and polymerisation [59,111]. If the reaction is conducted under aqueous conditions, the probability of dimerization has been reported to increase further due to hydrophobic packing of the fulvene molecules [120–121].

### Intramolecular cycloadditions

Whilst not as widely reported as intermolecular cycloaddition reactions, there are some interesting reports regarding the intramolecular cycloaddition of fulvenes, summarised in Table 1.

**Table 1:** Structures and cycloaddition notation of fulvene intramolecular cycloadditions.

Fulvene precursor	Cycloaddition product	Cycloaddition	References

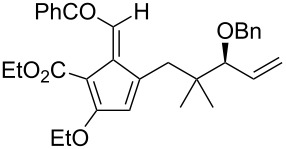	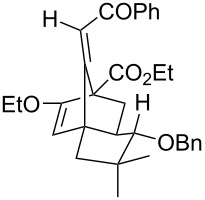	[4 + 2]	[122]
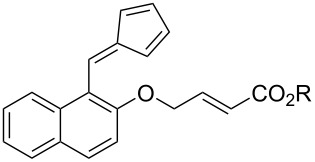	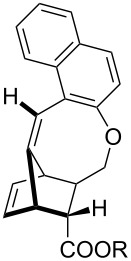	[4 + 2]	[91,119]
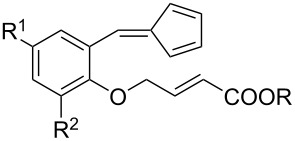	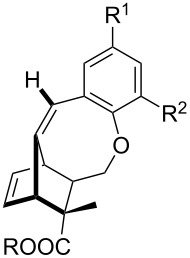	[4 + 2]	[91]
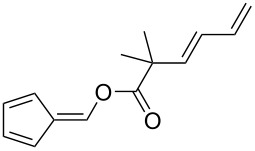	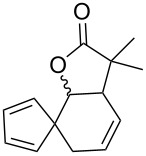	[4 + 2]	[123]
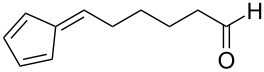	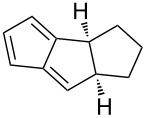	[6 + 2]	[85]
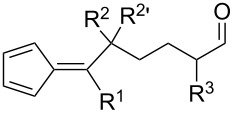	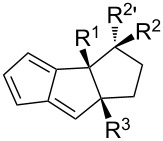	[6 + 2]	[124]
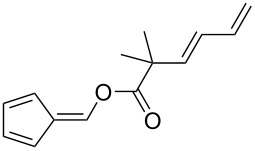	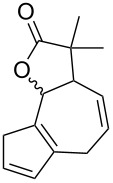	[6 + 4]	[123]
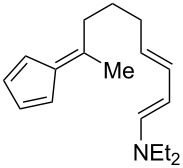	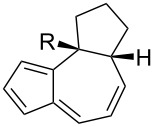	[6 + 4]	[125]
	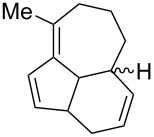	[4 + 2]	[125]
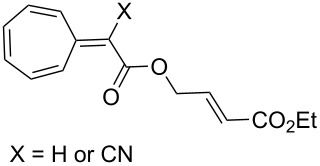	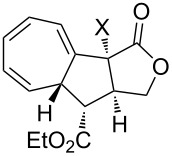	[8 + 2]	[126]
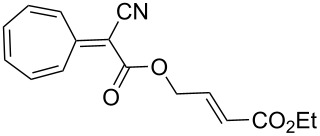	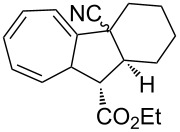	[8 + 2]	[126]

**Scheme 10:**
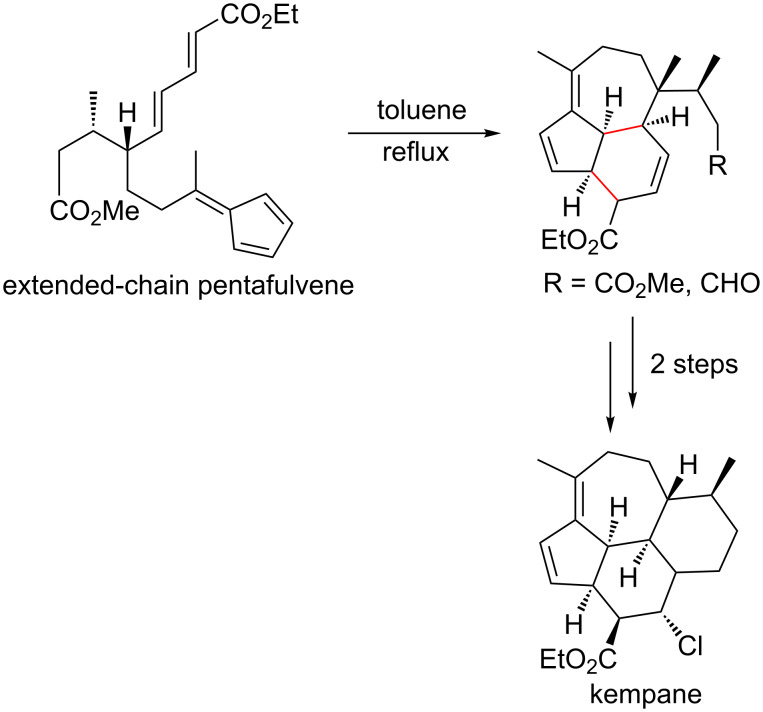
Simplified reaction scheme for the formation of kempane from an extended-chain pentafulvene [127].

For the intramolecular cycloadditions of pentafulvenes, the fulvene has been reported to react as both diene and dienophile depending on the reacting partner in the structure [91,119,127]. For example, pentafulvenes tethered to various dienes have been employed as precursors to various polycyclic ring systems, including kigelinol, neoamphilectane and kempane skeletons, which can be formed in a stereospecific manner depending upon the tether length of the extended pentafulvene chain, and the role of the fulvene in the reaction (diene or dienophile) [127]. In these examples, kigelinol and neoamphilectane are of great interest in biomimetic and natural product chemistry, as they exhibit antitrypanosomal [128–129] and antimalarial [130] activity, respectively. Soldier nasute termites use secretion of tetracyclic kempane skeletons as a defence mechanism [131], so their complete synthesis would invite further characterisation of the termite species. In a comprehensive study by Hong et al., precursor skeletons to kigelinol and kempane (Scheme 10) polycyclic ring systems were synthesised using DACs with extended-chain pentafulvenes, in 5 and 9 steps, respectively [127]. Progress has also been made towards synthesis of a neoamphilectane skeleton, but requires further optimisation to obtain the desired products.

**Scheme 11:**
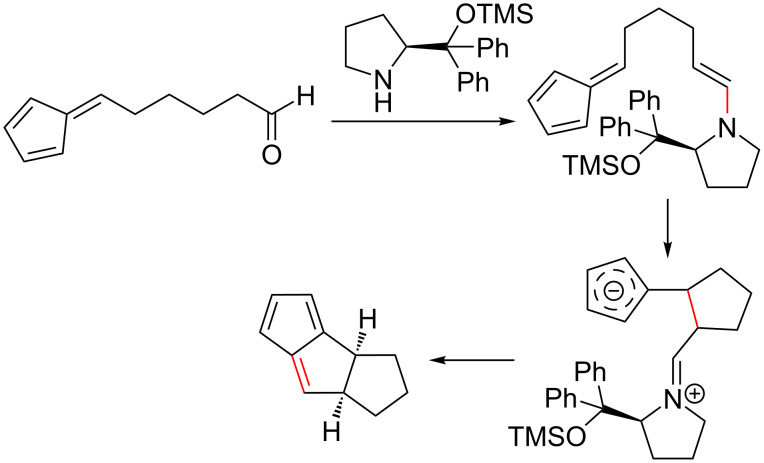
The enantioselective (>99% ee), asymmetric, catalytic, intramolecular [6 + 2] cycloaddition of fulvenes as reported by Hayashi et al. [85].

A versatile organocatalytic, enantioselective intramolecular cycloaddition reaction was reported by Hayashi et al. for the synthesis of various tricyclopentanoids from pentafulvenes with δ-formyl groups tethered to the exocyclic C6 position via structurally distinct spacers [85]. The intramolecular [6 + 2] cycloaddition was found to occur between the fulvene and an enamine generated through the reaction of the formyl group with the organocatalyst, diphenylprolinol silyl ether. Variation of the spacer structure provided access to a range of triquinane derivatives (Scheme 11), an important precursor in biomimetic and natural products [85].

**Scheme 12:**
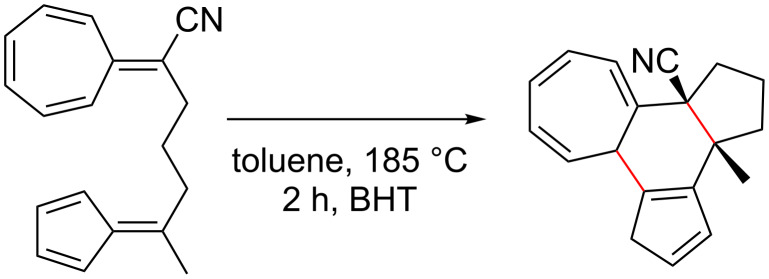
Intramolecular [8 + 6] cycloaddition of the heptafulvene-pentafulvene derivative [22,27].

Heptafulvenes have also been documented to react in intramolecular cycloaddition reactions [22,25,27]. Liu et al. synthesised an unsymmetric heptafulvene molecule containing a pentafulvene moiety (Scheme 12), which consequently underwent a [8 + 6] cycloaddition to diastereoselectively form a complex tetracycle [22,27].

### Intermolecular cycloadditions

Intermolecular cycloadditions of fulvenes have been studied using a wide range of different reactant partners to provide a varied array of different and often complex polycyclic scaffolds. The choice of reactant partner often determines the type of cycloaddition and how the fulvene will behave, and have been summarised in Table 2.

**Table 2:** Fulvene intermolecular cycloadditions with various reactant partners.

Fulvene	Fulvene component	Cycloaddition	Reactant partner	ref

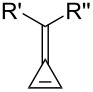 triafulvene	2π	[2 + 2]	enamines	[10]
			cyclic amines	[11]
		[4 + 2]	cyclopentadienes	[1,12]

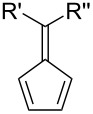 pentafulvene	2π	[1 + 2]	carbenes	[31,86,88]
		[2 + 2]	ketenes	[132–134]
			alkynes	[135–137]
			2,4,6‐triphenylpyrylium‐3‐olate	[138]
			dichloroketenes	[139–140]
		[3 + 2]	nitrones	[65,141–142]
			nitrile oxides	[143–144]
			3-oxidopyrylium betaine	[75,145]
			acylnitrones	[146]
			thiocumulenes	[79]
			Lewis acid mediated	[147]
			carbonyl ylides	[105]
			3-methyl-2,4-dipheny1-1,3-oxazolium-5-olate	[148]
			1,3-diphenylnitrilimine	[149]
		[4 + 2]	cyclopentadiene	[17,109,150–151]
			fulvenes	[17,24,137]
			tetrazines	[48]
			azirines	[57]
			halogenated dienes	[152]
			*o*-xylylenes	[153]
			dienes	[114,117,150,154–155]
			*o*-benzoquinones	[89,156–165]
			o-thioquinones	[166]
			2,4,6‐triphenylpyrylium‐3‐olate	[138]
			isobenzofurans	[77]
			diketones	[167]
			cyclopentadienones	[151]
			tetracyclic systems	[168]
			quinone methides	[87,169]
			coumalic esters	[170]
			tropone	[107]
			thiocarbonyl ylide	[171]
			Lewis acid catalysed	[172]
			mesoionic dithiolones	[76]
			1,3-oxazolium-5-olates	[173]
			benzonitrile oxide	[73]
			alkynes	[174]
		[8 + 2]	3-ethoxycarbonyl-2*H*-cyclohepta[*b*]furans	[18–19]
			fulvenes	[21,24,28]
			3-methoxycarbonyl-2*H*-cyclohepta[*b*]furan-2-one	[23]
			tropothione	[78]
	4π	[4 + 2]	fulvenes	[24]
			maleimides (including maleic anhydride)	[55,71,92,117,150,175–184]
			*p*-benzoquinones	[60,117,175]
			diphenylnitrone	[65]
			carboranes	[72]
			alkynes	[92,117,178,183,185]
			cyclopentadienone	[151]
			tetracyclic systems	[168]
			2,2-bis(trifluoromethyl)-1,1-dicyanoethylene	[186]
			alkenes	[117,177,187–194]
			cyclopentadiene	[177]
			2-chloroacrylonitrile	[178]
			triazoline-3,5-diones	[195–196]
			benzynes	[197–199]
			dienamines	[200]
		[4 + 3]	carbenes	[86,88]
			maleic anhydride	[117]
			2-oxyallyl cations	[100,201]
			1,3-diphenylnitrilimine	[149]
		[4 + 4]	*o*-benzoquinones	[158]
		[6 + 4]	*o*-benzoquinones	[89]
			tropone	[107]
	6π	[6 + 2]	alkynes	[136–137,202]
			4-methyl-1,2,4-triazoline-3,5-dione	[195]
			alkenes	[97]
			1-isopropenylpyrrolidine	[203]
		[6 + 3]	3-oxidopyrylium betaine	[75,90,145,204–207]
			2-oxyallyl cations	[100,201]
			carbenes	[208–209]
			isocyanoacetates	[93]
			iminoesters	[82]
			azomethine ylides	[81,83–84]
			*p*-benzoquinones	[97,210–211]
			azirines	[212]
			*N*-alkylidene glycine esters	[213]
			hydrazonyl chlorides	[214]
			indoanilines	[210]
		[6 + 4]	cyclopentadienes	[17,109]
			fulvenes	[17,24]
			tetrazines	[48]
			3-phenyl-2,2-dimethyl-2*H*-azirine	[57]
			*o*-benzoquinones	[158–161,165,215]
			*o*-xylylenes	[153]
			isobenzofuran	[77]
			azulene-indols	[216]
			coumalic esters	[170]
			tropone	[106–107,217]
			benzonitrile	[73]
			chlorooxime	[98]
			α-pyrone	[99]
			butadienes	[102,218–219]
			thiophene dioxides	[220–224]
			dienamines	[200,220,225]
			thiadiazole 1,1-dioxides	[226]
			mesoionic compounds	[94]
		[8 + 6]	fulvenes	[27]
			tropone	[112]
	10π	[10 + 4]	aziridinocyclobutane	[112]

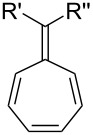 heptafulvene	2π	[4 + 2]	cyclohepta[*b*]furans	[18–19]
			1,3-diphenylthiazolo[3,4-*a*]benzimidazole	[20]
			fulvenes	[21,28]
			dienamines	[26]
			styrenes	[227]
	3π	[3 + 2]	hexane	[14]
	4π	[4 + 2]	fulvenes	[24]
			dienamines	[26]
		[6 + 4]	fulvenes	[24]
			dienamines	[26]
	8π	[8 + 2]	cycloheptatrieneFe(CO)3	[13]
			fulvenes	[24]
			styrenes	[227]

**Scheme 13:**
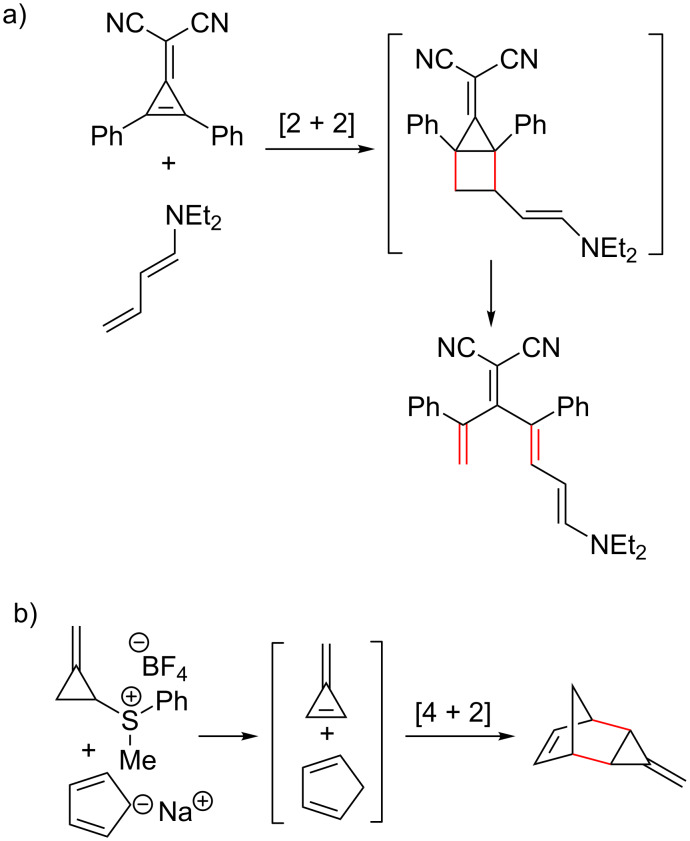
Reaction scheme for (a) [2 + 2] cycloaddition of 1,2-diphenylmethylenecyclopropene and 1-diethylamino-1,3-butadiene and (b) [4 + 2] cycloaddition of an in situ-generated triafulvene with cyclopentadiene.

Albeit one of the less documented fulvene classes (likely due to their extreme sensitivity [4,11–12]), triafulvenes have been reported to participate in both [2 + 2] [10–11] and [4 + 2] [12] cycloadditions. For the former, the reaction of an aminodiene with a triafulvene initially resulted in the formation of a [2 + 2] cycloadduct, and an energetically strained 4-membered ring inevitably undergoes subsequent ring-opening (Scheme 13a) [10]. During the [4 + 2] cycloaddition, the triafulvene could only be generated in situ from methyl(2-methylenecyclopropyl)(phenyl)sulfonium tetrafluoroborate (Scheme 13b).

As pentafulvenes are the most commonly studied fulvenes, it follows that there is a great deal of literature surrounding their reactivity in cycloaddition reactions. Due to conjugation, they can function as 2π, 4π, 6π or 10π components (Table 2). This functionality is dependent both on the other reactant partner, and the electronic effects of the fulvene substituents [96–99,153,156]. As an example, in [4 + 2] cycloadditions, fulvenes will participate as 4π components (diene), provided they are more electron-rich than the reactant partner [71,163,183].

**Scheme 14:**
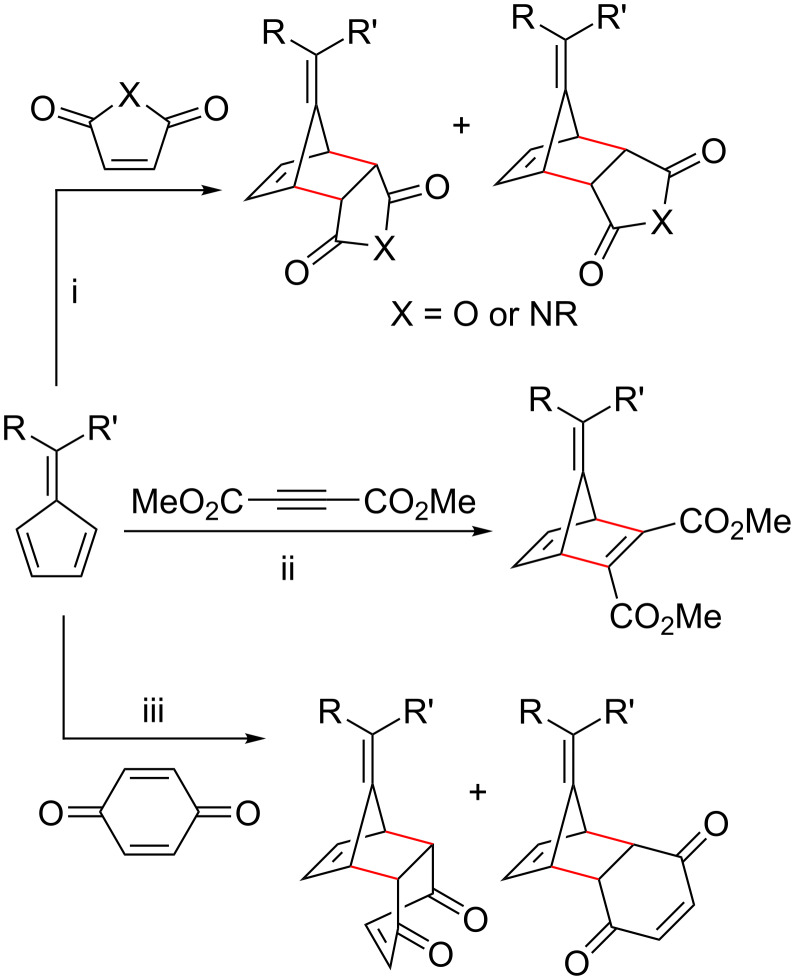
Diels–Alder cycloaddition of pentafulvenes derivatives participating as dienes with (i) maleimide derivatives, (ii) dimethyl acetylenedicarboxylate (DMAD) and (iii) p-benzoquinones.

Pentafulvenes show dual capabilities in DACs, with documented examples of them functioning as both dienes and dienophiles [55,114,150–151,154,159,174–176,227–229]. The exact nature of the fulvene moiety is dependent mostly on its substituents (e.g., EWG or EDG) relative to the other reactants [6,42,45,67,103,153,230]. Maleimides (including maleic anhydride) [55,71,92,96,150,176–177,179–184,186,192,229,231], dimethyl acetylenedicarboxylate (DMAD) and *p*-benzoquinone [60,150,159,164,175,211] derivatives [174,183,200,229] are often used as the complementary dienophiles (Scheme 14, reaction pathways (i), (ii) and (iii), respectively), as well as mono- and disubstituted acetylene derivatives, such as methyl propiolate [229] and dibenzoylacetylene [150].

**Scheme 15:**
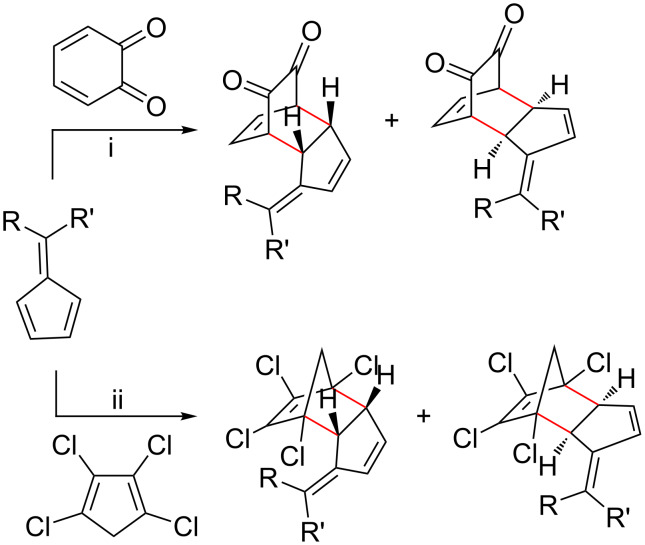
Generic schemes showing pentafulvenes participating as dienophiles in Diels–Alder cycloadditions with (i) *o*-benzoquinones and (ii) polyhalogenated cyclopentadienes.

Conversely, when the fulvene has an EWG attached, it is more likely to function as a dienophile in an inverse electron-demand Diels–Alder (iEDDA) reaction [153–154,156]. This requires the other reactant to have strong EDGs in order to function as a diene, otherwise fulvene dimerization becomes the preferred reaction, causing the formation of complex products. Examples of dienes that have previously been used include cyclic diketones (*o*-benzoquinones) (Scheme 15, reaction pathways (i)) [60,89,156–158,160–165,211,215,226], *o*-quinone methides [87], *o*-xylylenes [125,153], polyhalogenated cyclopentadienes (Scheme 15, reaction pathways (ii)) and 2-azadienes [152,172].

Regardless of the role of the fulvene moiety, the DAC is generally conducted in organic solvents at room temperature and under an inert atmosphere to prevent unwanted oxidations [24,55,94,114,150,152,159,166,172,174,176,183,186,227,229]. There are very few papers reporting the aforementioned reaction occurring in aqueous conditions [175] most likely as a result of the poor solubility of fulvene derivatives in water [175].

Although the stereochemistry of DACs can usually be predicted by the ‘endo rule’ [92,176,229,232], there are some exceptions, particularly when sterically-demanding fulvenes, such as norbornyl-fused fulvenes [229] or adamantilydenefulvene [174] are involved. In the literature, many cycloaddition reactions have been conducted with dimethylfulvene [52,97,106,118,133–134] or diphenylfulvene [20,103,114,133,163,180]. In each instance, the *endo* stereochemistry of the cycloadduct is dominant [91,176,180], indicating that the fulvene substituents in the exocyclic C6 position are too distal to impact the stereoselectivity [76,229].

**Scheme 16:**
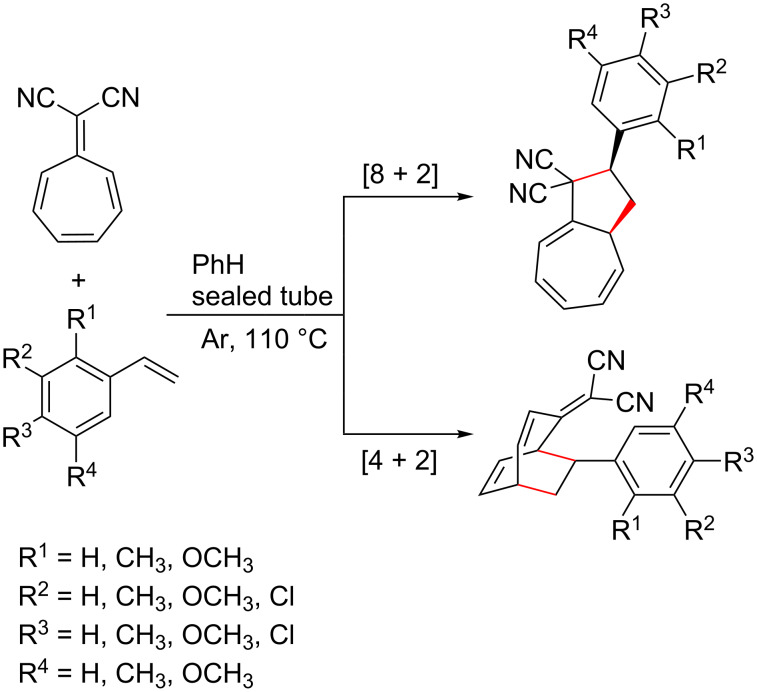
Reaction of 8,8-dicyanoheptafulvene and styrene derivatives to afford [8 + 2] and [4 + 2] cycloadducts in a 1:1 ratio [227].

There are documented cases of heptafulvenes [18–21,26,28,227] also participating in such reactions. However, Nair et al. reported that during cycloadditions of 8,8-dicyanoheptafulvene and styrene derivatives (Scheme 16), [8 + 2] and [4 + 2] adducts formed in approximately 1:1 ratio for each styrene variant tested, thus lowering the yield of the Diels–Alder adduct [227].

**Scheme 17:**
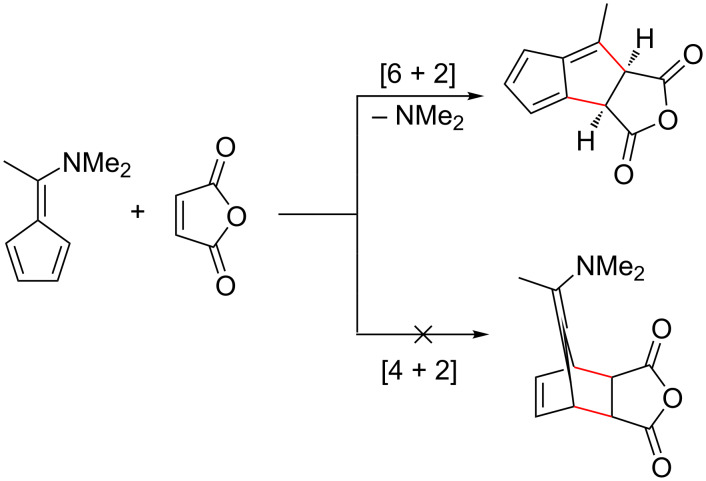
Reaction of 6-aminofulvene and maleic anhydride, showing observed [6 + 2] cycloaddition; the [4 + 2] cycloaddition is not observed [114].

Of particular interest is the reaction between 6-aminofulvenes and maleic anhydride. As previously reported, a fulvene reacting with maleimides (including maleic anhydride) generally results in a [4 + 2] cycloaddition (Scheme 14, reaction pathway (i)) [55,96,150,176,229]. However, when Houk et al. attempted to react a range of 6-aminofulvenes with maleic anhydride, a [6 + 2] cycloaddition was observed (Scheme 17) [114]. This unusual reactivity is hypothesised to be due to an increased electron density in the 6-aminofulvene π-system [96], which would increase the nucleophilic character, and stabilise the fulvene system (see dipolar forms in Scheme 1). Similar results have been observed by other groups [32,96,124,203].

Whilst many of the documented reactions focus on chemical synthesis and characterisation rather than applications, several synthetically interesting scaffolds have been synthesised, including products which exhibit biological activity, complex ligands in coordination chemistry, and several natural product skeletons (Table 3).

**Table 3:** Cyclic scaffolds prepared from fulvenes, grouped according to their applications.

Application	Product	ref

complex ligands	Fischer carbine complexes	[87,202,206–207]
	1,2-dihydropentalenes	[201]
	chromanes	[86]
	cyclopentachromenes	[96,165,209]
natural product skeletons	11-heterosteroids	[209]
	indans	[199]
	anislactones	[53,95]
	marrilactones	[95]
	hirsutate	[95]
	prostaglandins	[227]
	pyranopyrones	[167]
	pallambins	[185]
	iridoid monoterpenes	[231]
	aminocyclopentitols	[53]
	11-oxasteroids	[53]
	hirsutane	[53]
	histurane	[95]
	kigelinol	[125]
	kempanes	[125]
biologically active compounds	azairidoids	[82]
	pyrazoline	[59,232]
	azulenes	[18,23,93,97,100,121,151,168,198,217–219,223–224,233]
	dl-senepoxyde	[59]
	indenes	[85,99,206–207]
	pyrazoline	[31,47,212]
	pyrazolines	[147,232]
	diazepines	[158]
	quinoxalines	[155,158]
	tricyclopentanoids	[122]
	polycycclic cage systems	[166,173]
	eleven-membered carbocycles	[204]
	chromophores	[135]
	indenes	[91,145,208]
	carboranes	[71]
	cyclopentaoxazines	[96]
	azapolycycles	[194]
	tricyclic scaffolds	[89,184]
	pyridines	[92,152,210–211]
	pyrindines	[210–211]
	1,4-oxathiins	[164]
	iridoid monoterpenes	[231]
	dihydropyridines	[92]
	piperidines	[80,83]
	cyclooctanoids	[89,202–204,209]

### Applications of fulvene cycloadditions

#### Organic and natural product synthesis

A variety of organic molecules and natural products have been synthesised using fulvenes in cycloadditions (Table 3). Pentafulvenes appear to be the only fulvenes used in this approach, likely due to their relative stability compared to other members of the fulvene family, diverse cycloaddition chemistry, and easy access [42,45,64,67]. The synthesis of the listed organic molecules (Table 3) is generally successful, with high yields in almost all cases. However, some of these synthetic pathways are multistep [124], hence require optimisation for viability and large-scale production.

Similarly, pentafulvenes have been used as key reactants for the synthesis of natural products and their skeletons (Table 3). The complexity of these molecules requires extensive multistep pathways (ranging from 5–12 steps [127,187]), decreasing overall yields, and thus requiring further optimisation for commercial production. Narayan et al. developed a programmable enantioselective one-pot synthesis of molecules with eight stereocentres greatly improving the efficiency of natural product synthesis [83].

Each of these natural products are biologically active, hence their total synthesis will allow further characterisation of their reactivity and mechanisms of action.

#### Dynamic combinatorial chemistry

Dynamic combinatorial chemistry (DCC) is an emerging field with promising applications in drug discovery. DCC involves the generation of new molecules via reversible reactions of simple building blocks, referred to as a dynamic combinatorial library (DCL). As the reactions are reversible, several different structures are possible and the system exists in equilibrium. Upon the addition of an external surface (binding target), the equilibrium is altered and the product most stabilised through surface binding is amplified. Under optimal conditions, the desired molecule can be isolated in a high, preparative yield [233]. However, this is not always the case, and there are several factors that must be considered when designing a DCL. All components must be completely soluble, including the products. Failure to achieve this would cause irreversible precipitation of a product, and an inevitable shift in dynamic equilibrium.

**Scheme 18:**
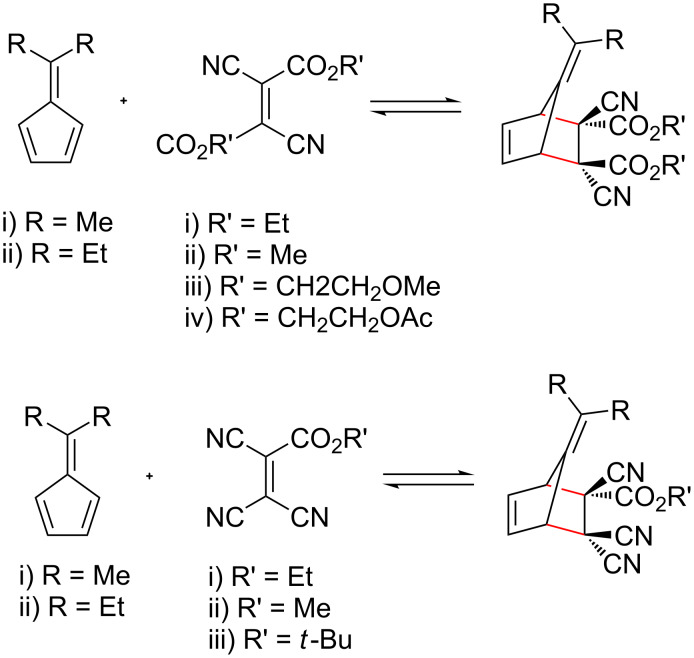
Schemes for Diels–Alder cycloadditions in dynamic combinatorial chemistry reported by Boul et al. Reactions occur between a pentafulvene and dicyanoethylenecarboxylate or tricyanoethylenecarboxylate [189].

Several types of reversible reactions have been successfully employed in the formation of DCL, including transesterification, peptide bond exchange, disulphide exchange, olefin metathesis and boronic ester formation [189,233]. Boul et al. recently investigated the application of fulvene DAC in DCC [189]. While the reaction is reversible, the retro-DAC generally only occurs at higher temperatures, which is not ideal. However, the combination of fulvenes and di- or tricyanoethylenecarboxylates was found to be reversible (and dynamic) under mild conditions at 25–50 °C (Scheme 18) [189]. At lower temperatures (−10 to 0 °C) the reaction was considerably slower, but overall suggests that certain fulvene DACs can be applied in DCC.

#### Materials chemistry

**Scheme 19:**
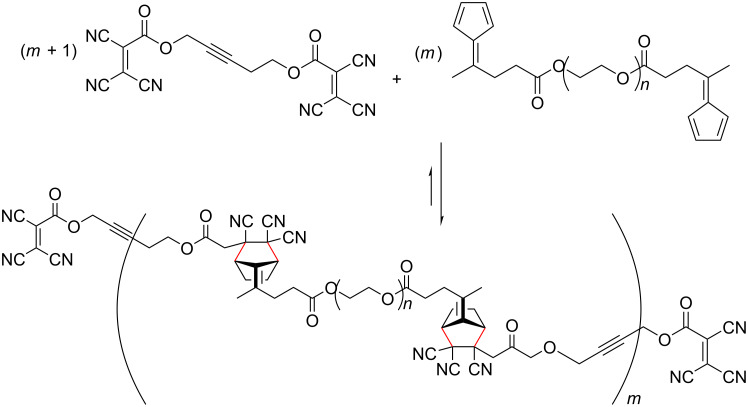
Polymerisation and dynamer formation via Diels–Alder cycloaddition between fulvene groups in polyethylene glycol bis(fulvene) and bis(tricyanoethylenecarboxylate) derivatives [190].

Despite their reactive nature, fulvenes have been successfully used in the formation of several materials, including dynamic polymers (dynamers) [190], hydrogels [191], and precursors to charge-transfer complexes [181,234–235]. Dynamers, referred to as dynamers, are a class of adaptive polymers formed through reversible covalent bonds or noncovalent interactions, allowing continuous modification through bond formation and/or breaking. This dynamic nature facilitates reorganisation through the exchange of building blocks, or incorporation of new substituents, even after the initial polymer has been formed [192]. The fulvene DAC is a good candidate for dynamer formation, as it is reversible at elevated temperatures [7,192]. A recent study by Reutenauer et al. developed dynamers using DAC of fulvenes (diene) and dicyanofumarate or tricyanoethylenecarboxylate (dienophile) (Scheme 19) [190]. The polymerisation (including the dynamic reversibility) was conducted at room temperature and the resulting polymers were processed as thin films. As a result of the dynamic nature of the Diels–Alder adducts, the films were shown to possess self-healing capabilities [190].

**Scheme 20:**
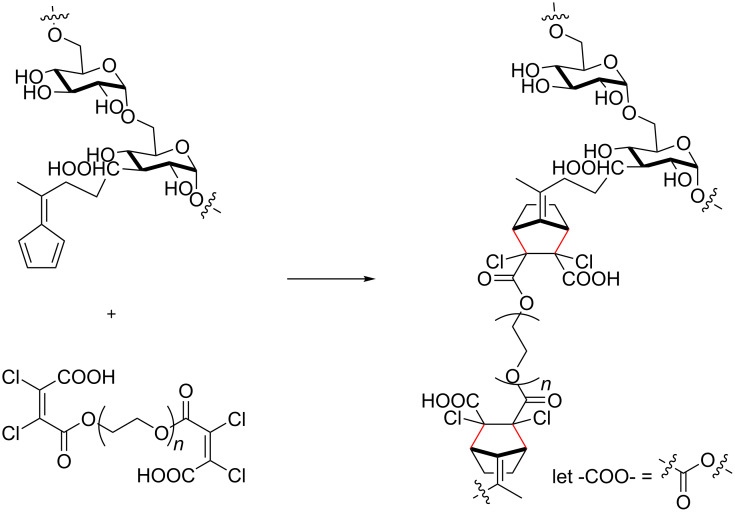
Preparation of hydrogels via Diels–Alder cycloaddition with fulvene-conjugated dextran and dichloromaleic acid-modified poly(ethylene glycol) [191].

Similarly, Wei et al. employed DAC to create a self-healing hydrogel using a polysaccharide functionalised fulvene as the polydiene. Initially, a fulvene derivative (4-(cyclopenta-2,4-dien-1-ylidene)pentanoic acid) was conjugated to dextran, and employed in DAC at 37 °C with a dichloromaleic acid-modified PEG derivative (Scheme 20) [191].

The formed hydrogels exhibited self-healing at physiological temperatures, as well as low levels of cytotoxicity against mouse fibroblast 3T3 cells [191]. With these characteristics in mind, the outlook for these hydrogels having therapeutic applications is promising, with further optimisation [236].

**Scheme 21:**
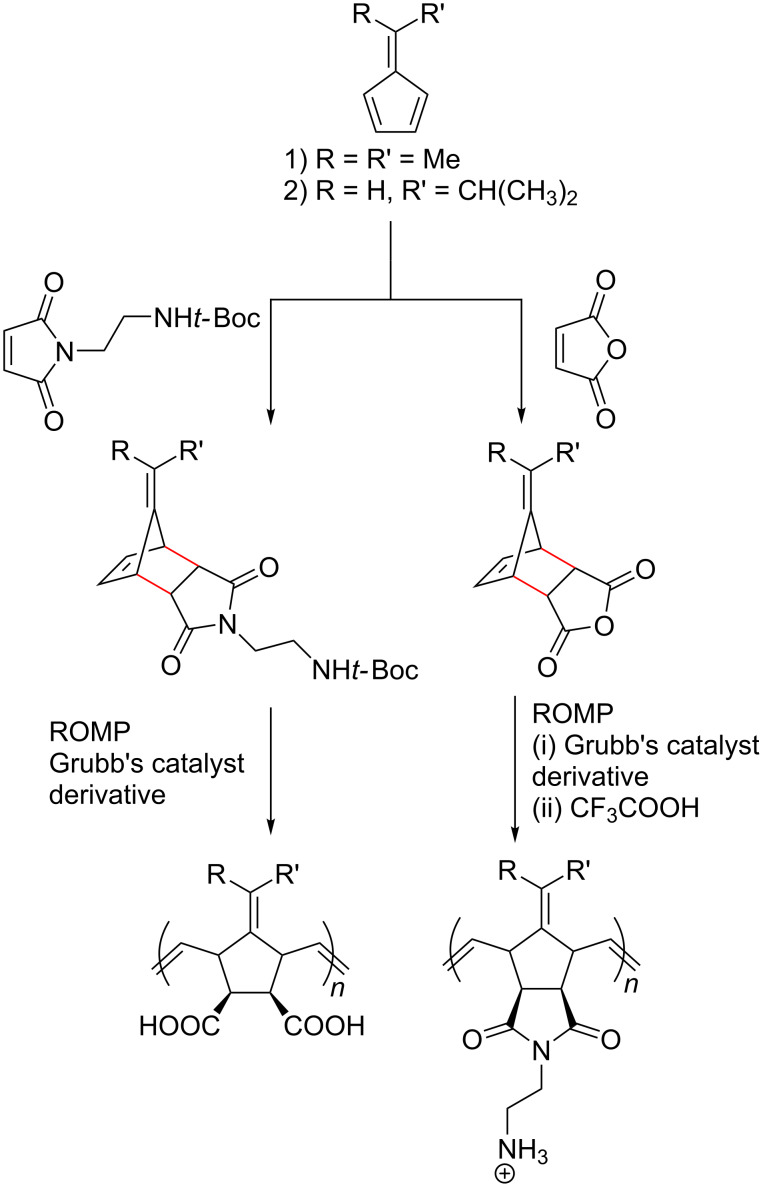
Ring-opening metathesis polymerisation of norbornene derivatives derived from fulvenes and maleimides to furnish facially amphiphilic polymers.

Pentafulvenes have also been used to prepare monomers for ring-opening metathesis polymerisation (ROMP) to generate facially amphiphilic polymers [182,235,237–238]. Ilker et al. employed the DAC between alkyl pentafulvenes and maleic anhydride to initially prepare norbornene anhydride monomers that could be further functionalised to afford norbornene imide monomers (Scheme 21) [105,237]. ROMP of the monomers, followed by deprotection yielded facially amphiphilic polynorbornenes that displayed lipid membrane disruption and antimicrobial activities [237–238].

The facially amphiphilic polynorbornenes with pendent ammonium groups were found to disrupt negatively charged phospholipid unilamellar vesicles at low concentrations (5 µg/mL), and in a dose and molecular weight dependent fashion, indicating their potential antimicrobial properties. Further studies revealed that co-polymerisation of norbornene imide monomers with different alkyl groups provided optimal antimicrobial properties and low haemolytic activities [237].

## Conclusion

This review provides an account of the properties and application of fulvene cycloaddition reactions. The interest in fulvenes due to their unique electronic properties and ability to undergo multiple highly selective cycloaddition reactions have fuelled advances in organic and natural product synthesis, dynamic combinatorial chemistry and materials science, including dynamers, hydrogels and charge transfer complexes. The recent advances show that potential applications for fulvene cycloaddition reactions are varied and wide in scope. We believe this review will lead to increased interest in these fields, and others yet to be investigated.
